# Hepatic SIRT3 Upregulation in Response to Chronic Alcohol Consumption Contributes to Alcoholic Liver Disease in Mice

**DOI:** 10.3389/fphys.2019.01042

**Published:** 2019-08-13

**Authors:** Yue Ma, Hui Chai, Qinchao Ding, Qianyu Qian, Zhaoyuan Yan, Bin Ding, Xiaobing Dou, Songtao Li

**Affiliations:** ^1^College of Life Sciences, Zhejiang Chinese Medical University, Hangzhou, China; ^2^Laboratory Animal Center, Zhejiang Academy of Medical Sciences, Hangzhou, China; ^3^Molecular Medicine Institute, Zhejiang Chinese Medical University, Hangzhou, China; ^4^College of Basic Medicine and Public Health, Zhejiang Chinese Medical University, Hangzhou, China

**Keywords:** sirtuin 3, autophagy, liver injury, hepatotoxicity, ALD

## Abstract

**Background:**

Alcoholic liver disease (ALD) is a type of chronic liver disease caused by chronic ethanol overconsumption. The pathogenesis of ALD is complex and there is no effective clinical treatment thus far. SIRT3 is an NAD^+^-dependent deacetylase primarily located inside mitochondria, and reports on the effect of chronic alcohol exposure on liver SIRT3 expression are scarce. This study aims to investigate the effect of chronic alcohol consumption on hepatic SIRT3 expression and its role in alcoholic-induced liver injury.

**Methods:**

Using the Lieber-DeCarli mouse model of ALD, we analyzed the regulation of SIRT3 and the effect of liver-specific knocking-down of SIRT3 on alcohol-induced liver injury. HepG2 and AML12 hepatocytes were employed to detect the biological function of SIRT3 on alcohol-induced hepatic cytotoxicity and its potential mechanism.

**Results:**

Chronic alcohol exposure led to hepatic SIRT3 upregulation and liver-specific SIRT3 knockdown alleviated alcoholic feeding-induced liver injury and lipid accumulation, which is associated with improved autophagy induction. In addition, autophagy induction contributed to the cytoprotective effect of SIRT3 knockdown on ethanol-induced hepatocyte cell death.

**Conclusion:**

In summary, our data suggest that hepatic SIRT3 upregulation in response to chronic alcohol exposure and liver-specific SIRT3 knockdown, induced autophagy activation further alleviating alcoholic-induced liver injury, which represents a novel mechanism in this process.

## Introduction

Alcoholic liver disease (ALD) is a type of chronic liver diseases caused by chronic ethanol overconsumption. It ranks among the major causes of morbidity and mortality due to liver diseases in the world, and affects millions of patients worldwide each year (O’Shea et al., 2010). The pathogenesis of ALD is complex and obviously multifactorial (Lawrence and Crab, 2001; Zakhari and Li, 2007). Excessive fat (triglycerides) deposition in the hepatocytes (hepatic steatosis) is the most common and earliest response of the liver to chronic alcohol consumption. Intracellular triglycerides accumulation increases the susceptibility of hepatocytes to the detrimental effects of “second hit,” primarily proinflammatory cytokines and oxidative stress, leading to the progression of steatohepatitis (Stephen et al., 2001; Reddy and Rao, 2005). Although much progress has been made during the past several decades, our understanding on the pathogenesis of ALD at cellular/molecular levels remains incomplete.

Autophagy, a self-digestive system involving degradation of dysfunctional/damaged intracellular proteins and organelles, is an evolutionarily conserved process in which autophagic substrates are sequestered in autophagosomes and delivered to lysosomes for degradation (Mizushima et al., 2008). Lipidation of microtubule-associated protein 1 light chain 3 (LC3) and the fusion of lysosomes with autophagosomes are critical steps in maintaining proper autophagic flux (Mariño and López-Otín, 2004). Accumulated evidence supports that autophagy plays a crucial role in mediating essential homeostatic functions in the liver and a dysregulated autophagy activation process contributes to the pathogenesis of various liver diseases (Ezaki et al., 2014; Madrigal-Matute and Cuervo, 2016). The effects of alcohol on hepatic autophagy activation remain ambiguous and seem to be both dose- and drinking pattern-dependent. Whereas acute ethanol treatment activated hepatic autophagy, chronic exposure to alcohol-containing liquid diet suppressed autophagy activation in the liver. Nevertheless, enhancing autophagy activation *via* chemical activators protect against alcohol-induced liver injury in both models, suggesting that autophagy plays a pathological role in ALD (Dolganiuc et al., 2012; Wang et al., 2015).

Sirtuins are a family of seven NAD^+^ dependent protein deacetylases (Haigis and Sinclair, 2010; Imai and Guarente, 2010). SIRT3 is one of the mitochondrial sirtuins that plays a predominant role in regulating mitochondrial protein (de)acetylation processes. Livers of SIRT3 knockout (KO) mice exhibit hyper-acetylation of mitochondrial proteins (Lombard et al., 2007). It has been well-established that SIRT3 activation promotes ATP production, beta-oxidation, and urea cycle activity, while suppressing reactive oxygen species (ROS) levels and cell death (Hirschey et al., 2010; Hallows et al., 2011). Although these effects can be derived from the direct deacetylation and subsequent activation of mitochondrial protein targets by SIRT3, it can also result from the activation of upstream regulators of mitochondrial function, including adenosine monophosphate–activated protein kinase (AMPK) and peroxisome proliferator–activated receptor gamma coactivator 1-alpha (PGC-1 alpha) (Palacios et al., 2009).

Although the beneficial effects of SIRT3 activation have been widely reported in many physiological and pathological conditions, SIRT3 exhibited both pro- and anti- apoptotic roles depending on different stimulus exposures and virous cell types (Schaffer, 2003; Lombard and Zwaans, 2014). We recently reported that SIRT3 overexpression in hepatocytes was indeed associated with autophagy suppression, which contributes to lipotoxicity in hepatocytes/liver induced by saturated fatty acids (Li et al., 2017). The present study aims to investigate the effect of chronic alcohol consumption on hepatic SIRT3 expression/activation and its role in alcoholic-induced liver injury. We demonstrate that chronic alcohol exposure leads to SIRT3 upregulation in the liver and liver-specific SIRT3 knockdown alleviates alcoholic-induced liver injury, which is associated with improved autophagy induction, suggesting that upregulated hepatic SIRT3 expression contributes to the pathogenesis of ALD.

## Materials and Methods

### Chemicals

Alanine aminotransferase (ALT) assay kit, aspartate aminotransferase (AST) assay kit, triglycerides (TG) assay kit and hepatic cholesterol (TC) assay kit were purchased from Nanjing Jiancheng Bioengineering Institute (Jiancheng, Nanjing, China). Other chemicals were obtained from Sigma-Aldrich (Sigma-Aldrich, St. Louis, MO, United States).

### Animal Model and Experimental Protocol

The Lieber-DeCarli mouse model of ALD was employed as previously described (Ding et al., 2017). Male C57BL/6 mice weighing 25 ± 0.5 g (mean ± SD) were housed in the animal center of the Zhejiang Traditional Chinese Medical University. The mice were divided into three groups (*n* = 10 per group): pair-fed (PF) group, alcohol-fed/AAV8-control group and alcohol-fed/AAV8-SIRT3 (AF + SIRT3 KD) group. The PF group were maintained on an isocaloric control liquid diet (Bioserv, Frenchtown, NJ, United States) for 5 weeks. AF group were fed *ad libitum* with an ethanol-containing Lieber-DeCarli diet (ethanol-derived calories were increased from 30 to 36% during the first 4 weeks, with a 2% increase each week) for 5 weeks. In comparison, mice in the AF + SIRT3 KD group were fed the same ethanol-containing Lieber-DeCarli diet as above and for liver-specific SIRT3 knock down, the animals were injected in the tail vein with recombinant adeno-associated viral (AAV) serotype 8 gene transfer vectors, bearing a liver-specific promoter combination with the mouse SIRT3 knock down target sequence (Cyagen Biosciences Inc., Guangzhou, China). AAV8 vectors were administered by tail vein injection at a dose of 1 × 10^12^ viral titer/ml in a total volume of 100 μl/mice at the beginning of the experiment. Recombinant AAV8 vectors target mouse SIRT3 (NM_022433.2) under the control of albumin promoter (a liver-specific promoter) were generated by Cyagen Biosciences Inc. (Guangzhou, China). A non-coding plasmid carrying only the albumin promoter was used to produce vector control particles and the alcohol-fed/AAV8-control group (AF + AAV8-control) animals were injected in the tail vein with AAV8-control particles. Food intake and body weight were recorded daily and weekly, respectively. Mice were sacrificed 5 weeks later; the mice were anesthetized with Avertin (250 mg/kg body weight) after 4 h of fasting. Plasma, liver, heart, muscle and epididymal fat pad samples were harvested for assays.

### Cell Lines and Culture Conditions

The human hepatoma cell line HepG2 and the non-hepatoma hepatocyte cell line Alpha mouse liver (AML)-12 were both obtained from the American Type Culture Collection (ATCC, Manassas, VA, United States). The alpha mouse liver (AML)-12 hepatocyte cell line was established from a mouse transgenic for human transforming growth factor α, and was obtained from the American Type Culture Collection, and cultured in Dulbecco’s Modified Eagle Medium/Ham’s Nutrient Mixture F-12, 1:1 (DMEM/F-12, Sigma-Aldrich, 051M8322) containing 10% (v/v) fetal bovine serum (Life technologies, 10099-141), 5 mg/ml insulin (Sigma-Aldrich, I9278), 5 μg/ml transferrin (Sigma-Aldrich, T8158), 5 ng/ml selenium (Sigma-Aldrich, 229865), 40 ng/ml dexamethasone (Sigma-Aldrich, D4902), 100 U/ml penicillin, and 100 μg/ml streptomycin (Life technologies, 15140-122) at 37°C in a humidified O_2_/CO_2_ (95:5) atmosphere (Li et al., 2014). HepG2 were cultured in Dulbecco’s Modified Eagle Medium (DMEM) containing 10% (v/v) fetal bovine serum, 100 U/ml penicillin, and 100 μg/ml streptomycin at 37°C in a humidified O_2_/CO_2_ (19:1) atmosphere.

### RNA Interference

Cultured cells were transfected with human SIRT3 siRNA (GenePharma, Shanghai, China) using ExFect 2000 (Vazyme, Nanjing, China) according to the manufacturer’s instructions. In the control group, cells were transfected with scrambled siRNA (GenePharma, Shanghai, China).

### Lactate Dehydrogenase (LDH) Assay

**Lactate dehydrogenase** release into the culture medium was used to determine cell viability. LDH activity was determined *via* electronic spectrophotometrically at 490 and 680 nm using a commercially available kit, as previously described (Song et al., 2004). The LDH assay kit was purchased from Thermo scientific (Thermo Fisher Scientific, Middletown, VA, United States).

### Histological Examination

At the time of killing, small pieces of liver tissue were reaped and fixed immediately in 4% paraformaldehyde. After paraffin embedding, 5 μm sections were deparaffinized in xylene and were rehydrated through a series of decreasing concentrations of ethanol. Sections were stained with hematoxylin and eosin. Oil red O staining was done using freshly isolated liver tissue which was fixed in 10% neutral buffered formalin at 4°C for 2 days, transferred to a 20% sucrose solution for 2 days and then frozen in Tissue-Tek. Frozen tissues were sectioned at 8 μm on a Microm cryostat set to −19°C and then air dried. In brief, rehydrated liver sections were stained with Oil Red O from 5% stock solution in isopropanol diluted at 3: 2 with distilled water for 20 min and counterstained with Mayer’s Hematoxylin (Turrens et al., 2011).

### Quantitative Real-Time Reverse Transcription (RT)-PCR

Total RNA extraction, reverse transcription, and real time PCR were performed as described previously (Song et al., 2008). Briefly, total RNA from liver tissue, was isolated with a phenol-chloroform extraction. For each sample, total RNA was reverse transcribed using a high-capacity cDNA reverse transcription kit (Takara Bio, Dalian, China). The cDNA was amplified in MicroAmp Optical 96-well reaction plates with a SYBR Green PCR Master Mix (Takara Bio, Dalian, China). Relative gene expression was calculated after normalization by a house-keeping gene (mouse or human 18S rRNA). Primers:

*SIRT3* Forward, 5′-TGCCAGCTTGTCTGAAGCA-3′,
*SIRT3* Reverse, 5′-GTCCACCAGCCTTTCCACA-3′,
*18S* Forward, 5′-ATACATGCCGACGGGCGCTG-3′,
*18S* Reverse, 5′-CGGCTCGGGCCTGCTTTGAA-3′.

### Western Blotting Analysis

Western blot analysis was performed as previously described (Song et al., 2008), and the following antibodies were used: Anti-SIRT3, Anti-Bcl2, Anti-Bax (Cell Signaling Technology, Danvers, MA, United States). Anti-LC3B (Sigma-Aldrich, St. Louis, MO, United States), Beta-actin antibody and GAPDH antibody (Boster, Wuhan, China).

### Analysis of Autophagic Flux

The autophagic flux was measured as previously described (Sarkar et al., 2009). Briefly, the cells were pretreated with chloroquine (CQ), an inhibitor of lysosome acidification, and followed by the indicated treatment. The autophagic flux was determined by detecting GFP-LC3 puncta using laser scanning confocal microscope (Nikon A1R, Japan) and LC3-II expression by Western blot, respectively. For GFP-LC3 fluorescence detection, cells were transiently transfected with recombinant adenovirus GFP-LC3 (Hanbio Biotechnology Co. Ltd., Shanghai, China). The total number of green puncta was detected by confocal microscope from at least 50 cells for each individual experiment after different treatment.

### Statistical Analysis

All data were expressed as mean ± SD. Statistical analysis was performed using a one-way analysis of variance (ANOVA) and further analyzed by Newman–Keuls test for statistical difference. Differences between treatments were considered to be statistically significant at *p* < 0.05.

## Results

### Chronic Alcohol Consumption Induces Fatty Liver and Liver Injury

**FIGURE 1:**
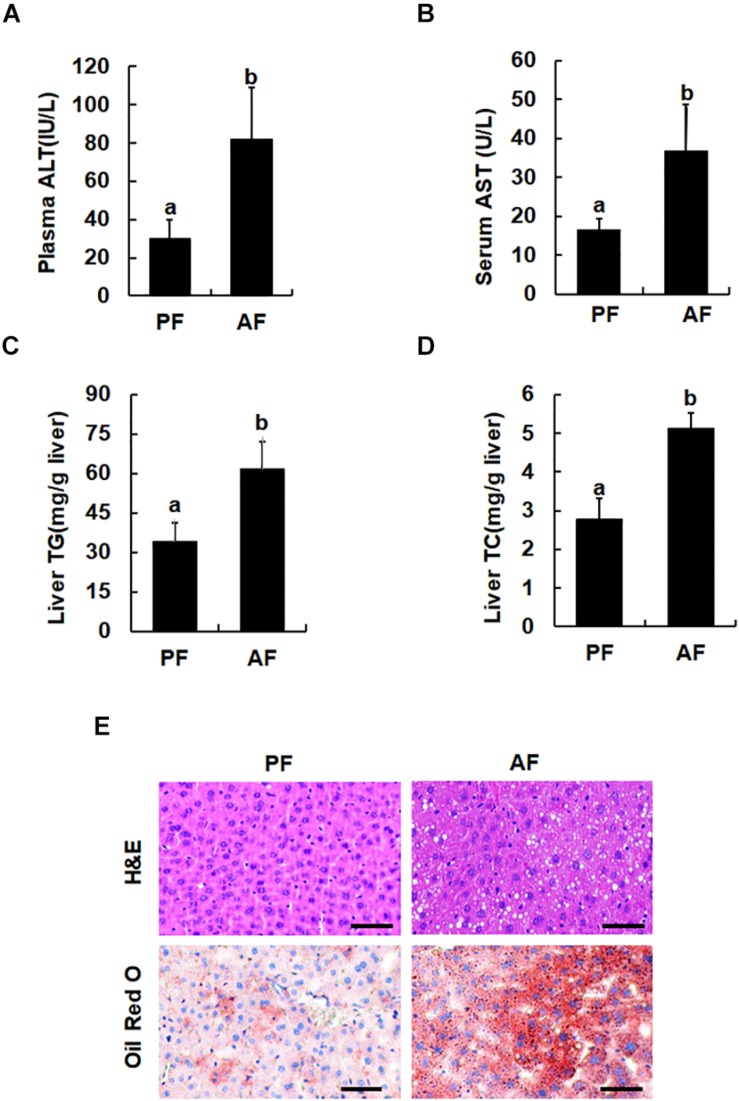
Chronic alcohol consumption induces fatty liver and liver injury. Male C57BL/6 mice were fed a control and ethanol-containing Lieber-DeCarli diet. PF, pair feeding; AF, alcohol feeding. **(A)** Plasma alanine aminotransferase (ALT) levels. **(B)** Serum Aspartate aminotransferase (AST) levels. **(C)** Hepatic triglyceride (TG) contents. **(D)** Hepatic cholesterol (TC) contents. Data are expressed as the mean ± SD (*n* = 10 mice per group). Bars with different letters (a, b) differ significantly (*p* < 0.05). **(E)** H&E staining and Oil Red of liver tissues. Bars mean 100 μm.

Male C57BL/6 mice (aged 8 weeks), fed the Lieber-DeCarli alcohol-containing liquid diet for 5 weeks, were used as an animal model of ALD. Consistent with previous reports, chronic alcohol exposure elevated plasma ALT and AST levels (Figures 1A,B). Compared with pair-fed animals, alcohol-fed mice manifested significantly increased hepatic TG and hepatic TC contents (Figures 1C,D), which were further confirmed by H&E and Oil Red O staining (Figure 1E).

### Chronic Alcohol Exposure Upregulates SIRT3 Expression in the Liver

**FIGURE 2:**
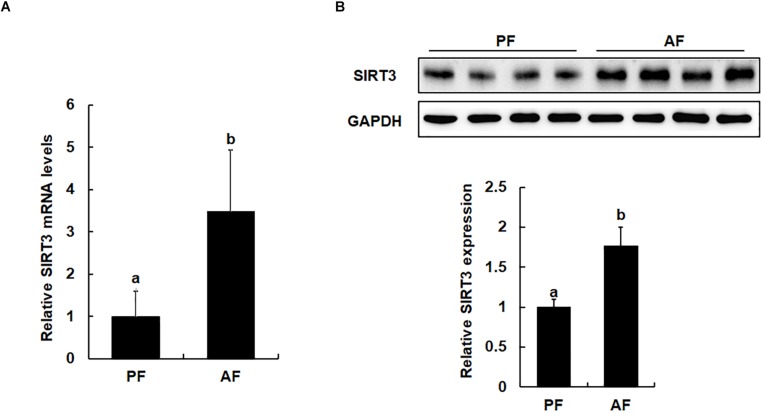
Chronic alcohol exposure is associated with upregulated liver SIRT3 expression. Male C57BL/6 mice were fed a control and ethanol-containing Lieber-DeCarli diet. PF, pair feeding; AF, alcohol feeding. All values are denoted as means ± SD Data (*n* = 8). **(A)** Real time PCR analysis of SIRT3 gene expression in liver of mice. **(B)** Protein expression of SIRT3 in the liver of mice as indicated by Western Blotting. Bars with different characters (a, b) differ significantly, *p* < 0.05.

The effects of long-term alcohol consumption on liver SIRT3 expression at both mRNA and protein levels were determined *via* qPCR and Western blot analysis. As shown in Figure 2, in comparison to pair-fed animals, feeding on alcohol for 5 weeks significantly increased hepatic SIRT3 mRNA levels (Figure 2A) and protein abundance (Figure 2B).

### Liver-Specific SIRT3 Knockdown Protects Against Alcoholic Liver Disease

**FIGURE 3:**
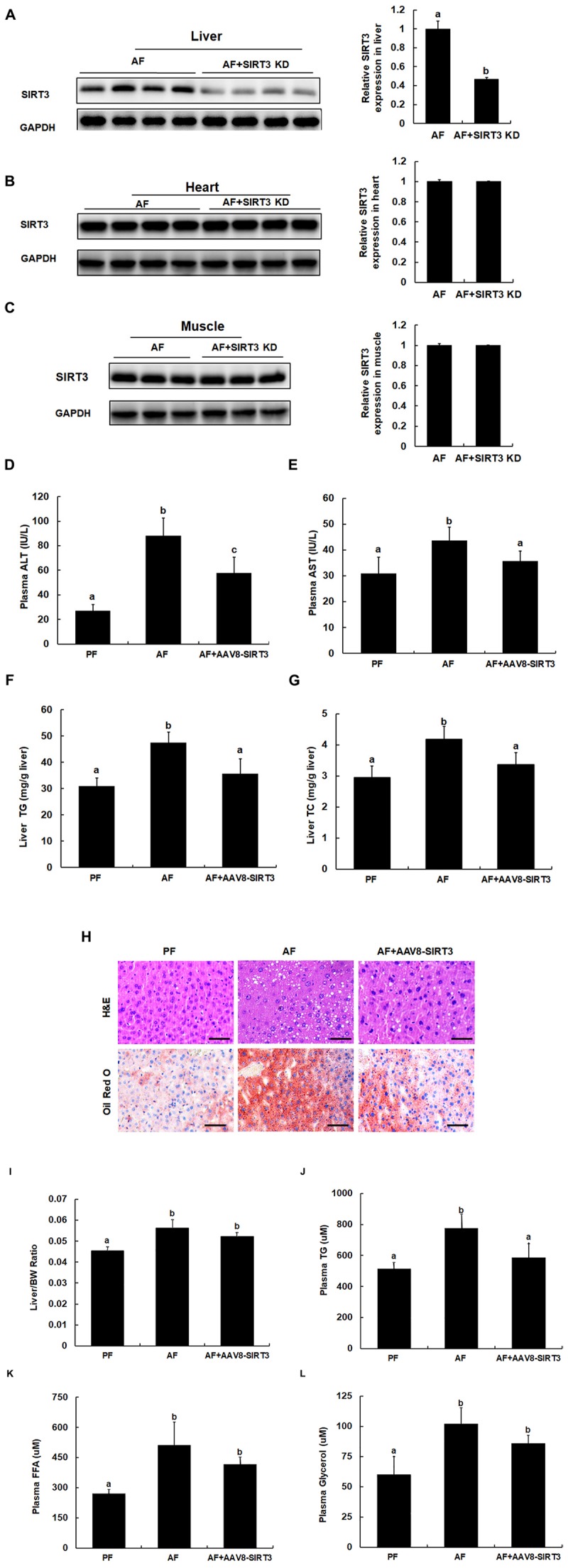
Liver-specific SIRT3 knockdown protects against alcoholic liver disease. Male C57BL/6 mice were fed a control and ethanol-containing Lieber-DeCarli diet. Total cellular lysates from liver tissues (*n* = 8) were subjected to immunoblotting assay for SIRT3. PF, pair feeding; AF, alcohol feeding mice were infected AAV8-control virus; AF + SIRT3 KD: alcohol-fed mice were infected with liver specific AAV8-SIRT3 KD. **(A)** SIRT3 protein expression in mice livers. **(B)** SIRT3 protein expression in mice hearts. **(C)** SIRT3 protein expression in mice muscles. **(D)** Plasma alanine aminotransferase (ALT) levels. **(E)** Plasma Aspartate aminotransferase (AST) levels. **(F)** Hepatic triglyceride (TG) contents. **(G)** Hepatic cholesterol (TC) contents. **(H)** H&E staining and Oil Red of liver tissues. Bars mean 100 μm. **(I)** Liver/body weight ratio. **(J)** Plasma triglyceride (TG) contents. **(K)** Plasma free fatty acids (FFA) contents. **(L)** Plasma glycerol contents. Data are expressed as the mean ± SD (*n* = 8 mice per group). Bars with different letters (a, b, c) differ significantly (*p* < 0.05).

To determine whether alcohol-induced SIRT3 upregulation in liver may contribute to the development of ALD, we established a mouse model with liver-specific SIRT3 knockdown by the AAV8-SIRT3 KD virus as described in the Section “Materials and Methods.” The liver-specific effect of the protocol was confirmed by Western blot detection of SIRT3 protein abundance. As shown in Figure 3, AAV8-SIRT3 KD virus infection resulted in a significant decrease of SIRT3 protein expression in the liver (Figure 3A), whereas in heart and muscle, SIRT3 expressions were not affected (Figures 3B,C). Importantly, when compared with alcohol-fed/AAV8-control animals, the mice with liver-specific SIRT3 knockdown manifested alleviated alcohol-induced liver injury, demonstrated by blunted increases of both ALT and AST levels (Figures 3D,E), lipids accumulation in the liver, evidenced by both biochemical assay (Figures 3F,G), and histological examination (Figure 3H). We also measured liver to body weight ratio (L/BW), plasma triglyceride (TG), plasma free fatty acids (FFA) and plasma glycerol contents. As shown in Figures 3I–L, in comparison to their counterparts, the mice with liver-specific SIRT3 knockdown improved these parameters in the setting of chronic alcohol exposure.

### SIRT3 Knockdown in the Liver Improves Hepatic Autophagy Activation

**FIGURE 4:**
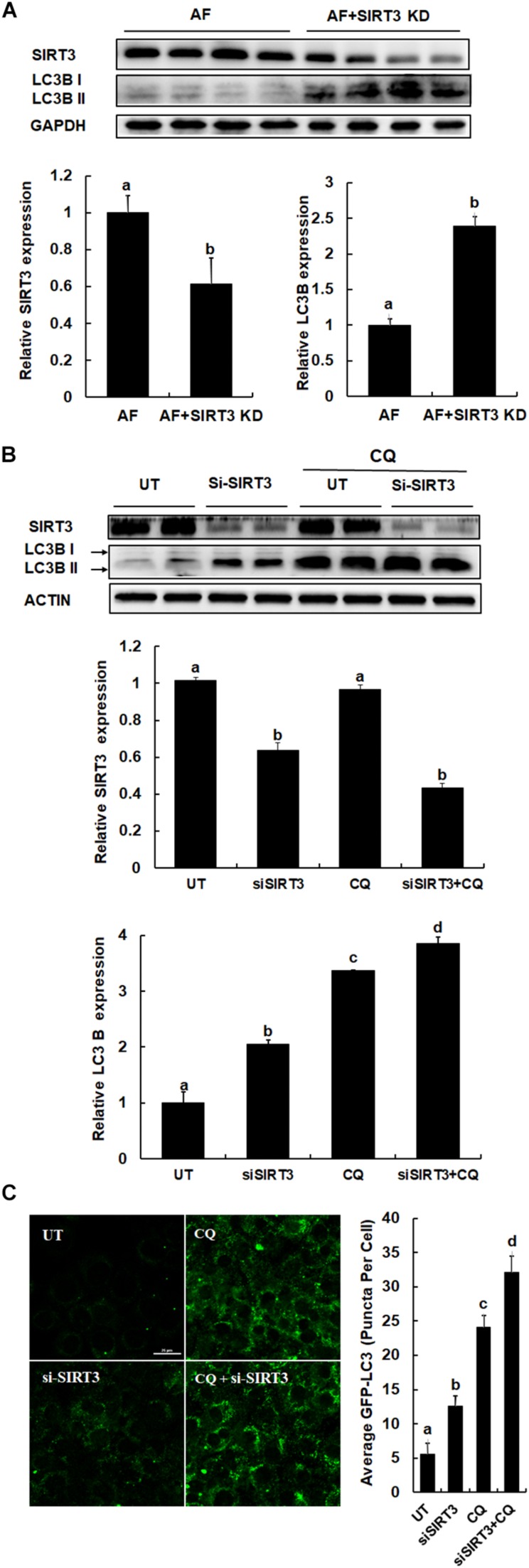
SIRT3 negatively regulates autophagy in hepatocytes. **(A)** LC3B and SIRT3 expressions were detected in liver specific SIRT3 KD and AF mice liver, respectively (*n* = 8). Quantification of LC3B and SIRT3 expression in liver. **(B)** HepG2 cells were transfected with siSIRT3 or scramble siRNA and treated with or without CQ (20 μm) for 12 h. Immunoblotting assay for SIRT3 and LC3B. Quantification of LC3B and SIRT3 expression in HepG2 cells. **(C)** HepG2 cells were co-transfected with recombinant adenovirus GFP-LC3 and siSIRT3 or scramble siRNA with or without CQ (20 μM) for 12 h. The total number of green puncta was counted. Bars mean 25 μm. AF, alcohol feeding mice were infected AAV8-control virus; AF + SIRT3 KD, alcohol-fed mice were infected liver specific AAV-SIRT3 KD. All values are denoted as mean ± SD from three or more independent batches of cells. Bars with different letters (a, b, c, d) differ significantly (*p* < 0.05).

We previously reported that SIRT3 is a negative regulator of autophagy in hepatocytes (20). To directly determine the effect of SIRT3 knockdown on liver autophagy induction in response to chronic alcohol exposure, we examined hepatic LC3-II conversion in the livers of alcohol-fed/AAV8-control and the liver-specific SIRT3 KD mice. As shown in Figure 4A, in comparison to alcohol-fed control animals, liver-specific SIRT3 knockdown mice manifested markedly increased hepatic LC3-II conversion. Similarly, in HepG2 cells, knocking-down SIRT3 *via* siRNA transfection led to increased LC3-II conversion (Figure 4B), and increased autophagic flux (Figure 4C).

### Autophagy Induction Contributes to the Cytoprotective Effect of SIRT3 Knockdown on Ethanol-Induced Hepatocyte Cell Death

**FIGURE 5:**
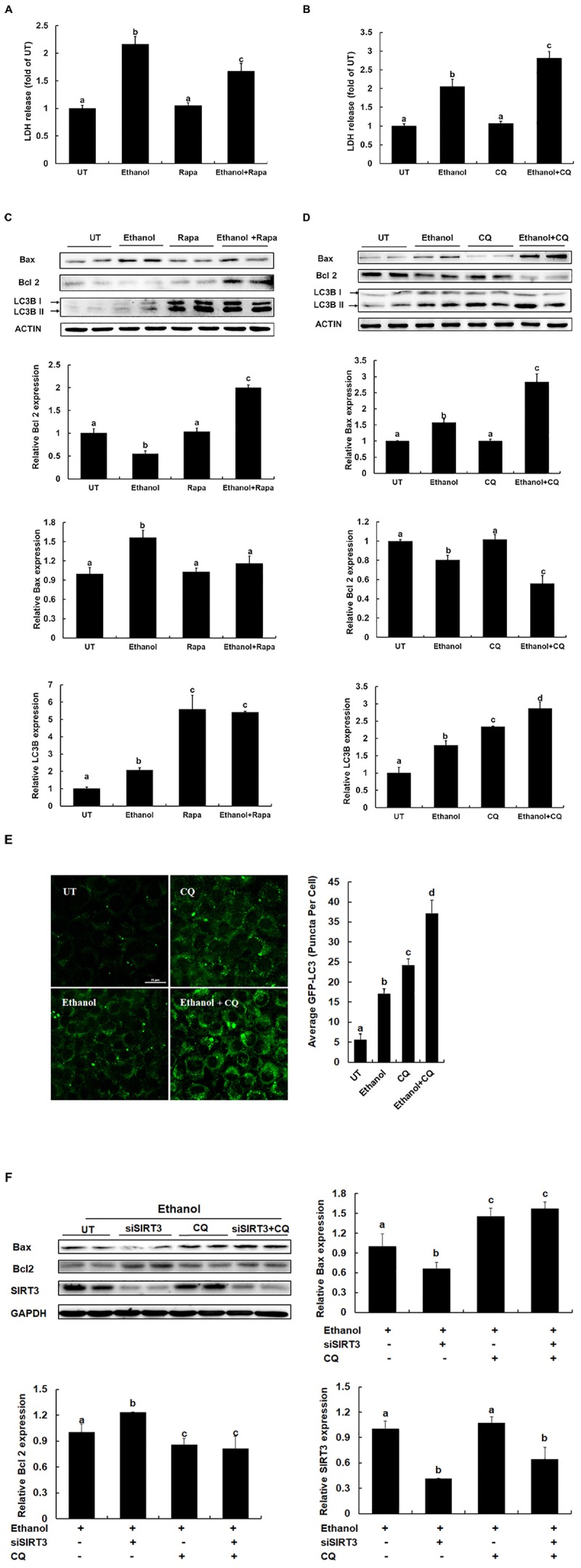
Autophagy inducer protects against alcoholic liver disease. **(A)** Autophagy inducer prevents Ethanol induced LDH release from AML12 cells. AML12 cells are treated with Rapamycin (100 nm) for 2 h before Ethanol (300 mM) addition. **(B)** Inhibition of autophagy promotes Ethanol induced LDH release from AML12 cells. AML12 cells are treated with CQ (20 μM) for 2 h before Ethanol (300 mM) addition. LDH release is measured 12 h later. **(C)** Autophagy inducer prevents apoptosis in ethanol-treated cells. AML12 cells were induced with Ethanol (300 mM) and treated with or without Rapamycin (100 nm) for 12 h. Immunoblotting assay for Bax, Bcl2 and LC3B. Quantification of Bax, Bcl2 and LC3B expression in AML12 cells. **(D)** Inhibition of autophagy promotes apoptosis in ethanol-treated cells. AML12 cells were induced with Ethanol (300 mM) and treated with or without CQ (20 μm) for 12 h. Immunoblotting assay for Bax, Bcl2 and LC3B. Quantification of Bax, Bcl2 and LC3B expression in AML12 cells. **(E)** AML12 cells were transfected with recombinant adenovirus GFP-LC3 and followed by CQ (20 μm) treatment for 2 h before Ethanol (300 mM) addition for 12 h. Total number of green puncta was counted. Bars mean 25 μm. **(F)** AML12 cells were transfected with siSIRT3 or scramble siRNA, induced with Ethanol (300 mM), and treated with or without CQ (20 μm) for 12 h. Immunoblotting assay for Bax, Bcl2, SIRT3, and LC3B. Quantification of Bax, Bcl2, LC3B, and SIRT3 expression in AML12 cells. All values are denoted as the mean ± SD from three or more independent studies. Bars with different letters (a, b, c, d) differ significantly (*p* < 0.05).

Ethanol is able to induce hepatocyte apoptosis and liver injury. Autophagy could be a major protective mechanism limiting ethanol toxicity. To understand the effect of autophagy in hepatocytes, the effect of autophagy *via* rapamycin and CQ treated on ethanol-induced AML12 cells was determined. As shown in Figure 5, autophagy inducer prevents ethanol induced cell death in AML12 cells, evidenced by a significant reduction of LDH release in comparison to ethanol alone treatment (Figure 5A), inhibition of autophagy promotes ethanol induced cell death in AML12 cells, evidenced by an increase of LDH release in comparison to ethanol alone treatment (Figure 5B). Furthermore, the intracellular anti-apoptotic gene Bcl2 expression was increased after the autophagy inducer was treated, and pro-apoptotic gene Bax was decreased when autophagy was significantly activated (Figure 5C). On the contrary, inhibition of autophagy led to decreased expression of Bcl2, and a robust increase of Bax (Figures 5D,E), suggesting that autophagy inducer protects against ethanol-induced liver injury. SIRT3 siRNA knockdown protect ethanol induced cell death in AML12 cells, whereas inhibition of autophagy led to the disappearance of this protection (Figure 5F), suggesting that SIRT3 knockdown induces autophagy activation and alleviating alcoholic-induced liver injury.

## Discussion

We previously reported that SIRT3 overexpression in hepatocytes was associated with suppressed autophagy induction, leading to aggravated palmitate-instigated lipotoxicity in hepatocytes and the liver (Li et al., 2017). The goal of this study was to examine how chronic alcohol consumption affects hepatic SIRT3 expression and to determine whether altered hepatic SIRT3 expression contributes to the pathogenesis of ALD. Using a well-established Lieber-DeCarli mouse model of ALD, we found that alcohol-induced hepatic pathological changes were concomitant with a significantly increased SIRT3 expression in the liver. Importantly, our data showed that specific SIRT3 knockdown in the liver alleviates alcoholic-induced liver injury, which was associated with improved autophagy induction. To our knowledge, this is the first report to show that the chronic alcohol consumption leads to upregulated SIRT3 expression in the liver, which contributes to the development of ALD.

Sirtuins belong to the class III histone/protein deacetylase family, catalyzing NAD^+^-dependent protein/histone deacetylation. Among the seven members of the sirtuin family (SIRT1-7), SIRT1, the founding member, is the most widely studied, whose dysregulation has been implicated in many pathological states and metabolic disorders. In the liver, the acetylation status, regulated by SIRT1, affect the activity of numerous transcription factors involved in lipid and glucose metabolism, including SREBP-1c, PGC-1α, and cAMP-responsive element-binding protein (CREB) regulated transcription co-activator-2 (CRTC2) (Liu et al., 2008). Several recent investigations have demonstrated that chronic alcohol drinking led to a reduced SIRT1 expression in the liver, resulting in an increase in lipogenic enzymes and a decrease in genes involved in fatty acid oxidation, collectively contributing to the development of fatty liver and liver injury (You et al., 2008a,b). It has been suggested that the depletion of intracellular NAD^+^ stores, due to alcohol consumption, plays a mechanistic role in alcohol-induced SIRT1 inhibition. SITR3 is the best-studied mitochondrial sirtuin and plays an important role in regulating mitochondrial functions (Haigis and Sinclair, 2010). Although it has been well-recognized that mitochondrial dysfunction plays a pathological role in ALD development, surprisingly, the reports on the effect of chronic alcohol exposure on liver SIRT3 expression are scarce. A study conducted by Fritz et al. (2012) demonstrated that chronic alcohol feeding of SIRT3 knockout mice was associated with a significant increase of mitochondrial protein acetylation when compared with wild type mice. Intriguingly, they did not provide data on how chronic alcohol consumption affects SIRT3 expression/activity in the liver (Fritz et al., 2012). Using a cell culture system, Kim et al. (2015) revealed that ethanol exposure downregulated SIRT3 protein expression within 3 days in AML-12 hepatocytes. In this study, we provided *in vivo* evidence demonstrating that chronic alcohol consumption is associated with a significant increase of SIRT3 expression in the liver.

Acetylation is an important post-translational modification process, playing a pivotal role in regulating protein functions. Chronic alcohol intake induces a global acetylation of proteins in the liver (Lombard et al., 2007). In line with many previous reports (Lombard et al., 2007; Hirschey et al., 2010; Hallows et al., 2011), our results (unpublished data) support that chronic alcohol exposure is associated with a significant increase of acetylated mitochondrial protein abundance (hyper-acetylation) in the liver. Given the fact that SIRT3 is a major mitochondrial deacetylase (Lombard et al., 2007) and a previous report suggesting that SIRT3-deficient mice manifest mitochondrial protein hyper-acetylation in the liver, it seems paradoxical that, in the current study, both SIRT3 upregulation and mitochondrial protein hyper-acetylation were observed in the liver of mice chronically exposed to an alcohol-containing diet (Palacios et al., 2009; Hirschey et al., 2011). It is worth noting although the beneficial effects of SIRT3 have been widely reported using mice with germline ablation of SIRT3, paradoxically, neither muscle-specific nor liver-specific SIRT3 knockout mice manifest any overt metabolic phenotype under either control (chow diet) or nutrient stress (high-fat diet) conditions, despite a marked global hyperacetylation of mitochondrial proteins (Fernandez-Marcos et al., 2012), suggesting that SIRT3 could be dispensable for mitochondrial (dys)function when it is deleted in a tissue-specific manner, a scenario present in the current study. On the other hand, accumulated evidence supports a proapoptotic role for SIRT3. It has been reported that SIRT3 is able to induce growth arrest and apoptosis in both cancer cells and in non-cancer cell lines (Grubisha et al., 2006; Alhazzazi et al., 2011; Shulga et al., 2016). Although it remains to be clearly elucidated, the data presented in our current study suggest that alcohol-induced liver-specific SIRT3 upregulation induces hepatocyte cell death, potentially sharing certain mechanism(s) as previously reported. Our present study could not provide evidence to elucidate the underlying mechanism(s) behind this; however, it is rational to postulate that the effects of alcohol intake on hepatic mitochondrial protein acetylation status involves multiple mechanisms. Apparently, pathways promoting protein acetylation in mitochondria, such as increased acetyl-CoA concentration and enhanced activities of acetyltransferases, have preponderance over those promoting protein deacetylation, including mitochondrial sirtuins. Further research is warranted to better understand the cellular/molecular mechanisms implicated in this process.

Autophagy in general is considered to be a survival mechanism that occurs during transient starvation, energy depletion, or cellular stress (Mizushima, 2005). Accumulated evidence supports that autophagy plays a crucial role in the pathogenesis of alcohol-related organ damage (Donohue, 2009), however, the effects of alcohol drinking on hepatic autophagy function remain controversial and seem to be both dose- and drinking pattern-dependent. For example, it has been reported that autophagy was inhibited in a dose-dependent manner in mice fed the Lieber-De Carli diet for 4 weeks (Lin et al., 2013). In another report, AMPK inhibition and subsequent autophagic suppression were observed in the liver of mice chronically exposed to an alcohol diet (You et al., 2004). Consistent with these findings, chronic alcohol consumption inhibits autophagy in hepatocytes and induces apoptotic cell death in a rat model (Cho et al., 2014). In contrast to these findings, acute alcohol exposure was found to activate autophagy in cultured primary hepatocytes (Ding et al., 2010; Thomes et al., 2012), with oxidative stress being the major contributing factor in this activation (Thomes et al., 2013). Moreover, in an acute alcohol exposure mouse model, acute alcohol suppresses mTOR signaling and activates AMPK under oxidative stress conditions, thereby activating autophagy (Sid et al., 2013). Regardless of the differential effects of alcohol drinking on hepatic autophagy function, it is currently unequivocal that the enhancement of autophagy function protects against ALD and might be a potential therapeutic target for the treatment of this liver disease (Ding et al., 2010; Lin et al., 2013).

The observations on the effect of SIRT3 on autophagy activation are controversial. Multiple groups have reported that SIRT3 activation promotes autophagy and mitophagy in cardiomyocytes and other cell lines (Webster et al., 2013; Papa and Germain, 2014; Yu et al., 2017), while several recent studies, including ours, demonstrated that SIRT3 overexpression was associated with autophagy suppression (Li et al., 2017). The data obtained in the present study demonstrated that liver-specific SIRT3 gene silencing/knockdown further enhanced hepatic autophagy activation in the setting of chronic alcohol feeding and improved liver pathologies. These observations are consistent with our previous report (Li et al., 2017). Furthermore, using a cell culture system, we demonstrated that SIRT3 knockdown induced autophagy activation alleviates alcoholic-induced liver injury. It can be speculated that the role of SIRT3 in the regulation of autophagy may vary depending on the physiological context as it controls a diverse set of mitochondrial proteins that have different functions. How SIRT3 can produce opposing effects on autophagy and fatty liver pathologies in different studies will require further experimentation for resolution.

## Conclusion

In summary, our data suggest that hepatic SIRT3 overexpression in response to chronic alcohol exposure plays an important role in the pathogenesis of ALD and liver-specific SIRT3 knockdown induced autophagy activation alleviates alcoholic-induced liver injury, which represents a novel mechanism in this process. Our results provide evidence that hepatic SIRT3 may be a possible therapeutic strategy to mitigate the pathology associated with ALD.

## Data Availability

All datasets generated for this study are included in the manuscript and/or the supplementary files.

## Ethics Statement

This study was carried out in strict accordance with the recommendations from the Guide for the Care and Use of Laboratory Animals of the Chinese Association for Laboratory Animal Science. All animal care and protocols were approved by the Animal Care and Use Committee of the Zhejiang Chinese Medical University. All killings were performed under Avertin anesthesia, and efforts were taken to minimize animal suffering.

## Author Contributions

YM and HC participated in the design and coordination of the study, collected the data and participated in the data interpretation, and wrote the manuscript. QD, QQ, ZY, and BD participated in coordination of the animal study, data collection and analysis. XD and SL participated in the design and coordination of the study and reviewed and approved the final manuscript. All authors read and approved the final manuscript.

## Conflict of Interest Statement

The authors declare that the research was conducted in the absence of any commercial or financial relationships that could be construed as a potential conflict of interest.
